# Spectrum of *JAG1* gene mutations in Polish patients with Alagille syndrome

**DOI:** 10.1007/s13353-014-0212-2

**Published:** 2014-04-20

**Authors:** Dorota Jurkiewicz, Dorota Gliwicz, Elżbieta Ciara, Jennifer Gerfen, Magdalena Pelc, Dorota Piekutowska-Abramczuk, Monika Kugaudo, Krystyna Chrzanowska, Nancy B. Spinner, Małgorzata Krajewska-Walasek

**Affiliations:** 1 Department of Medical Genetics, The Children’s Memorial Health Institute, Al. Dzieci Polskich 20, 04-730 Warsaw, Poland; 2 Department of Gastroenterology, The Children’s Memorial Health Institute, Warsaw, Poland; 3 Department of Pathology and Laboratory Medicine, The Children’s Hospital of Philadelphia and The Perelman School of Medicine, The University of Pennsylvania, Philadelphia, PA USA; 4 Department of Child and Adolescent Psychiatry, Medical University of Warsaw, Warsaw, Poland

**Keywords:** Alagille syndrome, Diagnostic strategy, *JAG1* gene, *JAG1* point mutations, Large deletions

## Abstract

Alagille syndrome (ALGS) is an autosomal dominant disorder characterized by developmental abnormalities in several organs including the liver, heart, eyes, vertebrae, kidneys, and face. The majority (90-94 %) of ALGS cases are caused by mutations in the *JAG1* (*JAGGED1*) gene, and in a small percent of patients (∼1 %) mutations in the *NOTCH2* gene have been described. Both genes are involved in the Notch signaling pathway. To date, over 440 different *JAG1* gene mutations and ten *NOTCH2* mutations have been identified in ALGS patients. The present study was conducted on a group of 35 Polish ALGS patients and revealed *JAG1* gene mutations in 26 of them. Twenty-three different mutations were detected including 13 novel point mutations and six large deletions affecting the *JAG1* gene. Review of all mutations identified to date in individuals from Poland allowed us to propose an effective diagnostic strategy based on the mutations identified in the reported patients of Polish descent. However, the distribution of mutations seen in this cohort was not substantively different than the mutation distribution in other reported populations.

## Introduction

Alagille syndrome (ALGS, OMIM #118459) is an autosomal dominant disorder characterized by developmental abnormalities in several organs including the liver, heart, eyes, vertebrae, kidneys, and face (Alagille et al. 1987). The diagnosis of ALGS is based on the appearance of bile duct paucity with at least three of the major clinical features including: chronic cholestasis, cardiac disease, skeletal abnormalities, ocular abnormalities, renal anomalies, and characteristic facial features (Emerick et al. 1999). The diagnosis of ALGS is hampered by its highly variable expressivity despite almost complete penetrance (Dhorne-Pollet et al. 1994). ALGS is caused by mutations in the *JAG1* (*JAGGED1*; MIM# 601920) or the *NOTCH2* (MIM# 600275) genes (Li et al. 1997; Oda et al. 1997; McDaniell et al. 2006; Kamath et al. 2012). Both genes are involved in the Notch signaling pathway. The *JAG1* gene encodes a cell surface ligand, whereas the *NOTCH2* gene encodes one of the four human Notch receptors. The *JAG1* gene is located within chromosome 20p12 and contains 26 exons encoding a conserved transmembrane protein. The JAG1 protein contains several evolutionarily conserved motifs, including a signal peptide, a DSL domain (Delta/Serrate/Lag2), 16 epidermal growth factor (EGF)-like repeats, a cysteine-rich region (CR), and a transmembrane domain (Lindsell et al. 1995). Mutations in the *JAG1* can be identified in around 90 % of clinically diagnosed individuals with ALGS (Warthen et al. 2006). To date, over 440 different *JAG1* gene mutations have been identified in ALGS patients (Li et al. 1997; Oda et al. 1997; Krantz et al. 1998; Yuan et al. 1998, 2001; Crosnier et al. 1999, 2000; Onouchi et al. 1999; Pilia et al. 1999; Heritage et al. 2000, 2002; Colliton et al. 2001; Giannakudis et al. 2001; Röpke et al. 2003; Jurkiewicz et al. 2005; Warthen et al. 2006; Kamath et al. 2009; Guegan et al. 2012; Lin et al. 2012; Wang et al. 2012). Ten individuals with ALGS features carrying various mutations in the *NOTCH2* gene have been reported to date (McDaniell et al. 2006; Kamath et al. 2012).

The purpose of this study was to determine the spectrum of *JAG1* mutations in a group of 35 Polish ALGS patients. The additional aim of the study was to review all mutations identified so far in Polish patients (Giannakudis et al. 2001; Stankiewicz et al. 2001; Röpke et al. 2003; Jurkiewicz et al. 2005, 2006) and compare them with mutations described in ALGS patients from other populations.

## Materials and methods

### Patients

Molecular analysis was performed in a group of 35 patients. The group consisted of 22 new unrelated patients referred to the Medical Genetics Laboratory in the Children’s Memorial Health Institute (CMHI) who have not been reported before and 13 patients from 11 unrelated families referred to CMHI in whom *JAG1* mutations were not revealed in previous studies. The group of the patients without detected *JAG1* mutations was originally reported (Jurkiewicz et al. 2005, 2006) and has been included in the present study after re-evaluation of clinical data. The patients who had three or more of the major clinical features of ALGS were referred for genetic testing. All patients met the standard clinical diagnostic criteria for ALGS (Alagille et al. 1987), although not all individuals had a liver biopsy performed. The study was approved by the Bioethics Committee of the Children’s Memorial Health Institute in Warsaw. Informed consent was obtained from all participating patients and their legal representatives.

### Mutation detection and analysis

Blood samples were collected from patients and their family members and genomic DNA was extracted from peripheral blood leukocytes by use of standard procedures. The complete coding sequence of the *JAG1* gene (26 exons) was amplified by polymerase chain reaction (PCR) as previously described (Krantz et al. 1998; Colliton et al. 2001; Warthen et al. 2006). PCR products were evaluated by a combination of single strand conformation polymorphism (SSCP) analysis, which was carried out on a GenePhor system (GE Healthcare, UK) and bi-directional sequencing on an ABI 3130 or an ABI 3730 analyzer (Applied Biosystems, Foster City, CA, USA). Sequences of analyzed fragments were compared with the *JAG1* cDNA sequence (GenBank RefSeq: NM_000214.2). The numbering of the nucleotide changes that were revealed was based on the reference sequence with the A of the ATG translation initiation codon as nucleotide +1.

ALGS patients found to be negative for *JAG1* mutations by sequencing were screened for large deletions by multiplex ligation probe-dependent amplification (MLPA) using the SALSA MLPA kit P184 JAG1 (MRC-Holland, Amsterdam, the Netherlands) according to the manufacturer’s instructions. MLPA was performed with 200 ng of genomic DNA. Probe amplification products were run on the ABI 3730 DNA analyzer. Peak plots were visualized and normalized, and the dosage ratios were calculated using GeneMarker software v1.8 (Soft Genetics LLC, State Collage, PA, USA). Probe ratios below 0.67 were considered to indicate a deletion and if the ratio was above 1.33, a duplication was called. Samples from healthy control subjects were included in each assay. Analysis of samples showing evidence of alterations was repeated three times.

In three patients with MLPA alterations chromosomal microarray analysis was carried out using the Illumina Infinium SNP genotyping platform (Kamath et al. 2009). For other patients with abnormal MLPA aCGH analysis was performed using CGX3×720K or Human CGH 3 × 1.4 M WG v.1.0 arrays (Roche NimbleGen, Madison, WI, USA) according to the manufacturer’s instructions, data were analyzed using DEVA and Genoglyphix software (Roche NimbleGen, Madison, WI, USA). The patients analyzed with the SNP array were examined again with Roche NimbleGen arrays for consistency of results.

In five patients without *JAG1* mutations or large *JAG1* deletions, the 34 exons of the *NOTCH2* gene were sequenced using 36 primer pairs (McDaniell et al. 2006). The sequences generated were compared with the *NOTCH2* cDNA sequence (GenBank RefSeq: NM_024408).

DNA samples obtained from additional family members were screened following identification of a *JAG1* mutation in the proband.

The results from the analysis of the patients described in this study were then combined with results from *JAG1* analysis of 30 additional Polish patients previously reported in the literature (Giannakudis et al. 2001; Stankiewicz et al. 2001; Röpke et al. 2003; Jurkiewicz et al. 2005, 2006).

## Results

Mutations in the *JAG1* gene were found in 26 of the 35 ALGS patients (Table **1**). Mutations were identified in 18 of the 22 newly studied patients, and eight of the 13 patients that had been previously screened by SSCP. Twenty-three different mutations were identified including seven frameshift, five nonsense, three missense, two splice-site, and six gross deletions. Thirteen novel point mutations were detected. All of the identified *JAG1* point mutations map into the extracellular domain of the JAG1 protein and are distributed throughout the *JAG1* gene. Seventy-two percent (13/18) of all point mutations are localized in epidermal growth factor (EGF) repeat regions and 66 percent of them (seven frameshift and five nonsense) are expected to lead to premature termination codons. All missense mutations not described previously (p.Asn429Thr, p.Cys438Gly) were predicted to be probably damaging to the protein function in the in silico analyses performed by means of both PolyPhen2 and SIFT software.

**Table 1:** The *JAG1* gene mutations in Polish ALGS patients. All mutations identified in the current study as well as mutations detected in previous studies (Giannakudis et al. 2001; Stankiewicz et al. 2001; Röpke et al. 2003; Jurkiewicz et al. 2005, 2006) are shown

	Patient No.	Exon or intron	Mutation position ^a^	Predicted consequence	Protein domain ^b^	Origin ^c^	Phenotype ^d^	Reference
Frameshift	1	Ex 2	c.172_178del7	p.(Ala58fs)	SP-DSL	NM	L, H, E, F	Jurkiewicz et al. (2005)
	2	Ex 4	c.509delT	p.(Leu170fs)	SP-DSL	NM	L, H, E, F	Jurkiewicz et al. (2005)
	3	Ex 7	c.929delG	p.(Gly310fs)	EGF3	de novo	L, E, V, F	This study
	4	Ex 9	c.1197delG	p.(Val399fs)	EGF5	de novo	L, H, F	Jurkiewicz et al. (2005)
	5	Ex 12	c.1456_1457delAG	p.(Arg486fs)	EGF7	de novo	L, H, E, F	Jurkiewicz et al. (2006)
	6	Ex 12	c.1485_1486delCT	p.(Pro495fs)	EGF8	de novo	L, H, E, V, F	Jurkiewicz et al. (2005)
	7	Ex 14	**c.1736_1737delCA**	p.(Thr579fs)	EGF10	de novo	L, H, E, V^?^, F	This study
	8	Ex 14	c.1809_1810insTGGG	p.(Lys604fs)	EGF10	maternal	L, H ,E, F	Jurkiewicz et al. (2005)
	9	Ex 15	**c.1897delT**	p.(Cys633fs)	EGF11	de novo	L, H, V, F, R	This study
	10	Ex 16	c.2065_2066delTT	p.(Phe689fs)	EGF12	de novo	L, H, E, F	Röpke et al. (2003)
	11	Ex 17	c.2122_2125delCAGT	p.(Gln708fs)	EGF13	ND	L, H, E, F	Jurkiewicz et al. (2005)
	12	Ex 18	c.2250delC	p.(Pro750fs)	EGF14	de novo	L, H, V, F	Röpke et al. (2003)
	13	Ex 22	**c.2648delG**	p.(Cys883fs)	CR	de novo	L, H, E, V, F	This study
	14	Ex 22	**c.2651-2652insA**	p.(Gln884fs)	CR	de novo	L, H, E, F	This study
	15	Ex 23	c.2753delT	p.(Ile918fs)	CR	NM	L, H, E, F	Jurkiewicz et al. (2005)
	16	Ex 25	**c.3197_3198insC**	p.(Thr1066fs)	CR-TM	ND	L, H, E, V^?^, F, R	This study
	17	Ex 26	**c.3230_3231insT**	p.(Leu1077fs)	TM	ND	L, H, E, V, F	This study
Nonsense	18	Ex 2	c.142G>T	p.(Glu48Ter)	SP-DSL	paternal	L, H, E, F	Jurkiewicz et al. (2006)
	19	Ex 2	**c.246T>G**	p.(Tyr82Ter)	SP-DSL	paternal	L, H, E, V, F	This study
	20	Ex 2	c.383G>A	p.(Trp128Ter)	SP-DSL	de novo	L, H, F	Jurkiewicz et al. (2005)
	21	Ex 4	c.496C>T	p.(Gln166Ter)	SP-DSL	de novo	L, V, F	Jurkiewicz et al. (2005)
	22	Ex 5	c.703C>T	p.(Arg235Ter)	EGF1	de novo	L, H, E, V, F	This study^g^
	23	Ex 6	c.841C>T	p.(Gln281Ter)	EGF2	paternal	L, H, E, V, F	Jurkiewicz et al. (2005)
	24	Ex 9	c.1207C>T	p.(Gln403Ter)	EGF5	de novo	L, H, F	Jurkiewicz et al. (2005)
	25	Ex 10	**c.1325G>A**	p.(Trp442Ter)	EGF6	maternal	L, H, E, V, F	This study^g^
	26	Ex 13	c.1603C>T	p.(Gln535Ter)	EGF9	de novo	L, H, E, F	Jurkiewicz et al. (2005)
	27	Ex 18	c.2230C>T	p.(Arg744Ter)	EGF14	de novo	L, H, E, V, F	This study
	28	Ex 18	**c.2304C>A**	p.(Cys768Ter)	EGF14	maternal	L, V, F	This study
Splice site	29	IVS 2	c.388-1G>C	r.spl?	SP-DSL	de novo	L, H, E, F	Jurkiewicz et al. (2005)
	30	IVS 3	c.439 + 1G>A	r.spl?	SP-DSL	maternal	L, H, E, V, F	Jurkiewicz et al. (2006)
	31	IVS 3	c.439 + 1G>A	r.spl?	SP-DSL	de novo	L, H, E, F, R	Jurkiewicz et al. (2006)
	32	IVS 5	c.755 + 1G>A	r.spl?	EGF1	maternal	L, H, E, V, F	Giannakudis et al. (2001)
	33	IVS 6	**c.886 + 2_886 + 5del**	r.(spl?)	EGF2	maternal	L, H, E?, V?, F	This study
	34	IVS 10	**c.1348 + 1G>A**	r.spl?	EGF6	ND	L, E, F	This study
	35	IVS 11	c.1395 + 3A>G	r.(spl?)	EGF7	de novo	L, H, E, F	Jurkiewicz et al. (2006)
	36	IVS 24	c.3048 + 1_3048 + 2insG	r.(spl?)	CR-TM	de novo	L, H, E, V, F, R	Jurkiewicz et al. (2005)
	37	IVS 24	c.3048 + 5_3048 + 7delGTA	r.(spl?)	CR-TM	maternal	L, H, E, V, F, R	Röpke et al. (2003)
Missense	38 ^e^	Ex 2	c.359T>A	p.(Ile120Asn)	SP-DSL	maternal	L, H, E, V, F	Jurkiewicz et al. (2005)
	39 ^e^	Ex 2	c.359T>A	p.(Ile120Asn)	SP-DSL	maternal	L, H, E, F	Jurkiewicz et al. (2005)
	40	Ex 4	c.551G>A	p.(Arg184His)	SP-DSL	paternal	L, E, F, R	Giannakudis et al. (2001)
	41	Ex 4	c.551G>A	p.(Arg184His)	SP-DSL	paternal	L, H, E, V, F	Jurkiewicz et al. (2006)
	42	Ex 4	c.560G>A	p.(CysC187Tyr)	DSL	NM	L, E, V, F	Jurkiewicz et al. (2005)
	43	Ex 4	c.672G>T	p.(Trp224Cys)	DSL	de novo	L, H, F	Röpke et al. (2003)
	44	Ex 9	c.1156G>A	p.(Gly386Arg)	EGF5	ND	L, H, V, F	This study
	45	Ex 9	c.1156G>A	p.(Gly386Arg)	EGF5	de novo	L, H, E, F	This study
	46	Ex 10	**c.1286A>C**	p.(Asn429Thr)	EGF6	maternal	L, H, E, F	This study
	47	Ex 10	**c.1312T>G**	p.(Cys438Gly)	EGF6	maternal	L, H, E, F	This study
Large genomic rearrangements	48	Ex 20–23	c.2889-?_3376+?del		EGF15 – CR	de novo	L, H, E, V, F, R	This study^g^
	49	Ex 3–25	c.916-?_3999 + ?del		SP-DSL –CR-TM	ND	L, H, E, V, F, R	This study^g^
	50	Ex 1–25	53.9 kb deletion, breakpoints: 10,570,644-10,624,536	gene deletion	SP – CR-TM	de novo	L, H, E, F, R	This study^g^
	51	Whole gene	991 kb deletion, breakpoints: 9,818,619–10,810,007	gene deletion	all	de novo	L, H, E, F	This study
	52	Whole gene	2.26 Mb deletion, breakpoints: 9,272,721–11,534,825	gene deletion	all	de novo	L, H, F, R	This study
	53^e^	Whole gene	5.4 Mb deletion, breakpoints: 9,323,011–14,733,354	gene deletion	all	paternal	L, H, E, V, F, R	This study^g^
	54^e^	Whole gene	5.4 Mb deletion, breakpoints: 9,323,011–14,733,354	gene deletion	all	paternal	L, H, E, V, F	This study^g^
	55^f^	Whole gene	5.4 Mb deletion, breakpoints: 9,323,011–14,733,354	gene deletion	all	ND	L, H, E, V, F, R	This study^g^
	56	Whole gene	paracentric inversion of chromosome 20p12.2p13, insertion breakpoint between exons 5 and 6 of *JAG1* gene		EGF1	de novo	L, H, E, F	Stankiewicz et al. (2001)

^a^the *JAG1* sequence is that of the cDNA of the GenBank accession no. NM_000214.2; the nucleotide position at the A of the ATG translation start codon is denoted as nucleotide +1; novel point mutations appear in boldface print; chromosomal coordinates are given according to the GRCh37/hg19 assembly

^b^SP-DSL – region between signal peptide and DSL domain, DSL – Delta/Serrate/Lag2 domain, EGF – epidermal growth factor repeats domain, CR – cysteine-rich region, CM-TM – region between CR and TM, TM – transmembrane domain

^c^ ND – not determined, parent’s samples not available; NM – mutation not detected in mother’s sample, father’s sample not available

^d^main ALGS symptoms: L – liver, H – heart, E – eye, V – vertebrae, F – face, R – renal involvement, ? – the feature was not examined

^e^siblings

^f^cousin of the siblings no. 53, 54

^g^patients from previous studies eveluated again in this study

In one patient a substitution c.2048G>A (p.Arg683His) was identified but predictions from PolyPhen2 and SIFT described it as a benign change, thus it was considered a rare polymorphism. The change was not reported before. Molecular analysis of mother’s DNA did not reveal the change and the father’s DNA was not available.

The MLPA analysis revealed partial *JAG1* deletions in two patients (deletion of exons 3–25 and deletion of exons 20–23) and whole *JAG1* gene deletions in six patients (including two siblings and their paternal aunt). The exact size of the genomic alterations was further evaluated by aCGH. In one familial case of ALGS all three affected members carried the same deletion on chromosome 20, which was predicted to span at least 5.4 Mb. The deletions for patients no. 51 and 52 are predicted to span at least 991 kb and 2.26 Mb, respectively.

Sequencing of the *NOTCH2* gene in five patients in whom we did not identify a *JAG1* mutations or large deletion did not reveal any pathogenic alterations. We were unable to screen the *NOTCH2* gene in four *JAG1* negative patients.

## Discussion

Mutational analysis of the coding sequence of the *JAG1* gene in a cohort of 35 Polish ALGS patients has revealed 26 patients with mutations. Combined with the Polish ALGS patients previously reported in the literature (30 patients) we identify a cohort of *JAG1* positive Polish ALGS patients, with mutations in 56 patients coming from 53 families (Giannakudis et al. 2001; Stankiewicz et al. 2001; Röpke et al. 2003; Jurkiewicz et al. 2005, 2006) (Table **1**). Fifty different mutations were found. All of the identified mutations are localized in the extracellular domain of the JAG1 protein. Fifty-six percent of various point mutations map into epidermal growth factor (EGF) repeat regions. Sixty-five percent of point mutations (frameshift and nonsense mutations) are predicted to lead to premature termination codons. Most of the mutations were private, only three various mutations (c.439 + 1G>A, c.551G>A, c.1156G>A) were recurrent and each of them occurred twice in unrelated patients.

Over 440 various *JAG1* mutations have already been described in ALGS patients. The most common are frameshift mutations (49 %), followed by nonsense mutations (16 %), missense mutations (15 %), gross deletions and insertions (11 %), while the least frequent variants are splice site mutations (9 %) (Li et al. 1997; Oda et al. 1997; Krantz et al. 1998; Yuan et al. 1998, 2001; Crosnier et al. 1999, 2000; Onouchi et al. 1999; Pilia et al. 1999; Heritage et al. 2000, 2002; Colliton et al. 2001; Giannakudis et al. 2001; Röpke et al. 2003; Jurkiewicz et al. 2005; Warthen et al. 2006; Kamath et al. 2009; Guegan et al. 2012; Lin et al. 2012; Wang et al. 2012). The spectrum of *JAG1* mutations in the Polish ALGS patients presents a similar pattern to those from other groups, with only slight differences in the frequency of some types of mutations. As in previously reported studies, frameshift mutations were the most frequent (34 %), however, nonsense mutations and splice site mutations were more common than in other populations (22 % and 16 %, respectively). The frequency of missense mutations and large genomic rearrangements was almost the same as in other studies (14 % in both groups).

**Fig. 1:**
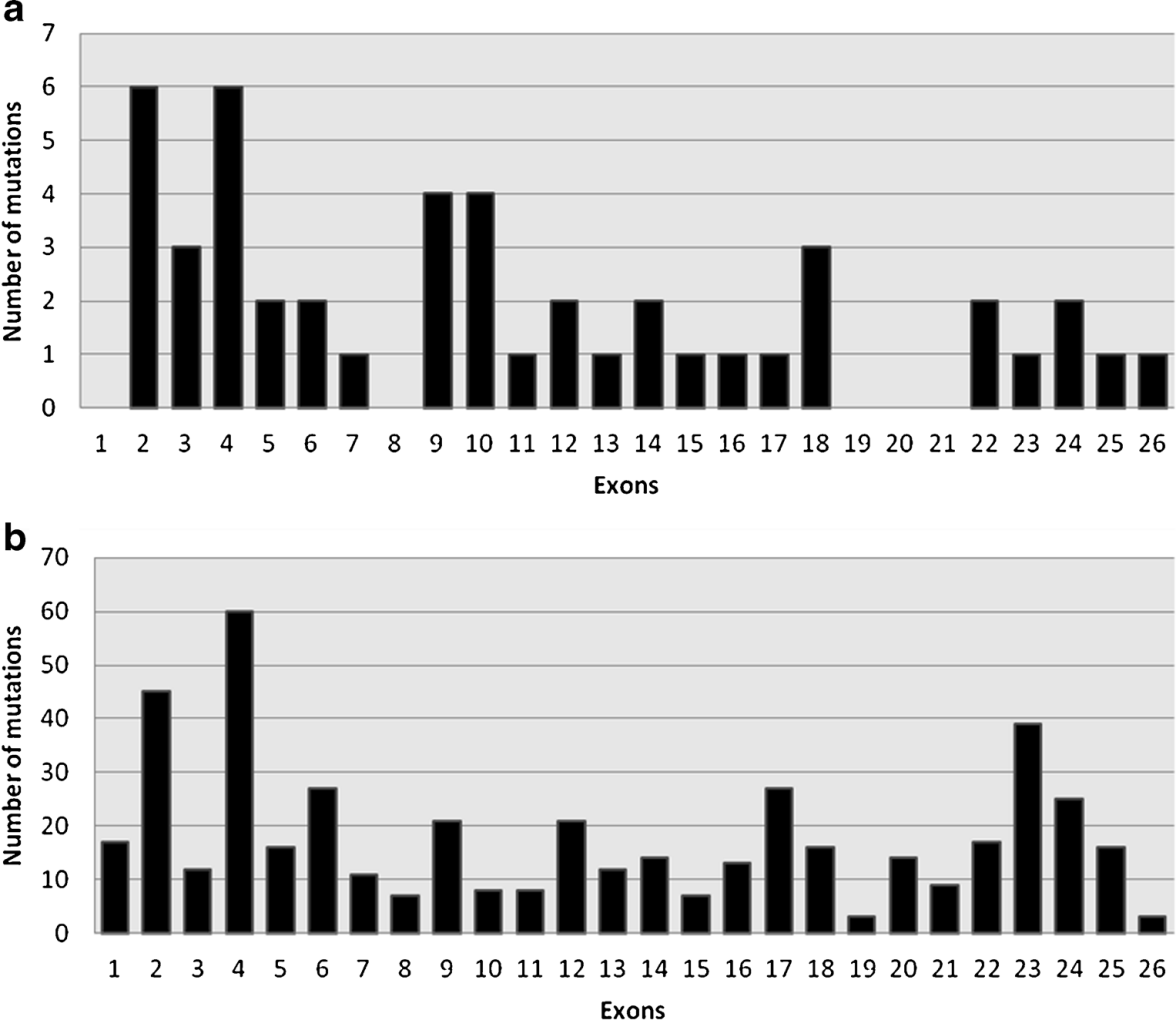
Distribution of *JAG1* mutations in ALGS patients. **a**: Polish cohort. **b**: Other reported populations

Mutations identified in the Polish cohort were spread over almost the entire *JAG1* gene except exons 1, 8, 19, 20, and 21 (Fig. 1). Forty-two percent of point mutations are found in four exons (2, 4, 9, 10) and 81 percent of point mutations are found in 12 exons (2, 3, 4, 5, 6, 9, 10, 12, 14, 18, 22, 24). No hot spot was found. In other reported populations *JAG1* mutations are also distributed along the whole gene, with 47 percent of point mutations localized in six exons (2, 4, 6, 17, 23, 24). Exons 2 and 4 are fragments of the *JAG1* gene with the highest number of mutations both in the Polish cohort and other populations.

Mutation screening of the *JAG1* gene in Polish patients in previous studies was mainly performed by SSCP followed by sequencing of selected fragments (Jurkiewicz et al. 2005, 2006). To see how the limitations of the SSCP technique decreases the mutation detection rate in this large gene, 13 patients from 11 unrelated families in whom mutations were not revealed were subject to *JAG1* sequencing. The MLPA analysis was also performed in this group of patients as it was not implemented in the previous studies. Only patients with classic presentation of ALGS were included in the mutation re-evaluation. The sequencing revealed nonsense mutations (c.703C>T, c.1325G>A) in two unrelated patients, consistent with single nucleotide substitutions having a higher likelihood of being missed by SSCP analysis. Moreover, MLPA screening has revealed gross deletions in six patients from four families that further underlines the usefulness and importance of that technique in ALGS diagnostics (Kamath et al. 2009; Lin et al. 2012). In five patients negative for *JAG1* mutations the *NOTCH2* gene sequencing revealed no pathogenic changes.

Overall, *JAG1* mutations were found in 53 Polish ALGS patients out of a group of 62 unrelated patients, and therefore, the mutation detection rate in the Polish cohort is 85 percent.

When patients from both this study and the literature are considered, analysis of parental samples was conducted in 43 families and revealed that mutations were inherited in 37 % of cases. In two families the presence of parental mosaicism was proved (Giannakudis et al. 2001), which should be taken into account in the diagnosis and genetic counseling. Analysis of parents for whom clinical data were available revealed that most of them were unaffected or presented only mild ALGS features such as the characteristic facial features or posterior embryotoxon. Three mothers had heart defects and two mothers had both heart and liver manifestations. Such a diverse clinical manifestation in individuals carrying the same primary disease causing mutation suggests a role for genetic modifiers of the clinical outcome.

**Fig. 2:**
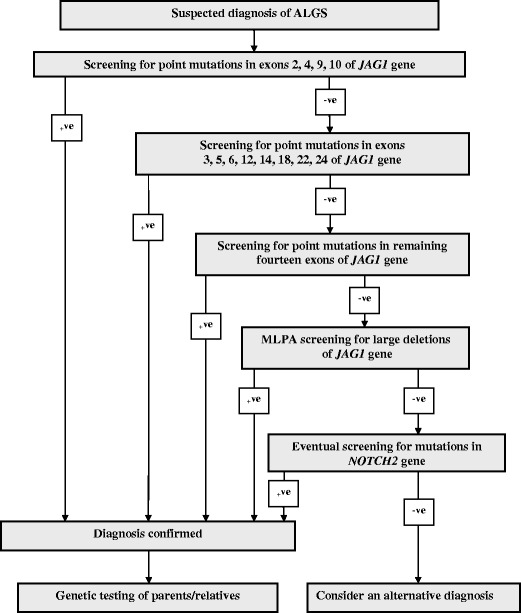
Diagram of proposed genetic investigations for suspected Polish ALGS patients

The large size of the *JAG1* gene makes diagnostics of ALGS labor intensive. However, analysis of the frequency and distribution of mutations along the *JAG1* gene enables us to propose an effective diagnostic strategy for Polish ALGS patients (Fig. 2). We suggest a tiered approach, with initial sequencing of the exons with the largest number of mutations (four or more), followed by a second tier (2–3 per exon), followed by sequencing of exons in which one or no mutation has been found so far. In patients negative for *JAG1* point mutations, MLPA screening for large deletions involving *JAG1* gene should be executed. The *JAG1* gene analysis might be completed by the *NOTCH2* gene screening, however the analysis of this gene seems to be of minor importance in the ALGS routine diagnostics as *NOTCH2* mutations have been reported so far only in ten patients with ALGS features (McDaniell et al. 2006; Kamath et al. 2012). The proposed strategy is based on methods commonly available in most laboratories, primarily Sanger sequencing. However, new molecular technologies are evolving rapidly fueled by the utilization of next generation sequencing (NGS) based tests (Pareek et al. 2011; Rabbani et al. 2014). This technology allows the simultaneous analysis of selected portions of the genome, such as several or many genes, or the whole exome or the whole genome. As new methods become more cost effective and accessible, the proposed diagnostic strategy will have to be updated.

In accordance with previous reports, no apparent correlation between genotype and phenotype was observed. In comparing the clinical phenotype of the Polish cohort with *JAG1* mutations to other cohorts, the liver phenotype occurs in 100 % and cardiac phenotype occurs in 89 % of the population (Table **1**), whereas both these features are present in over 94 % of other reported populations (Warthen et al. 2006; Lin et al. 2012). Renal involvement is observed in 21 % of Polish ALGS patients with *JAG1* mutations, while it occurs in 27–39 % of ALGS patients in other cohorts (Warthen et al. 2006; Kamath et al. 2001; Lin et al. 2012).

This study presents a comprehensive analysis of *JAG1* mutations in the cohort of Polish patients with ALGS. The review of all patients from the current and previous studies allows us to determine the genetic background of this population and to propose an effective diagnostic strategy for Polish ALGS patients.
